# A Chronological Overview of Using Deep Learning for Leukemia Detection: A Scoping Review

**DOI:** 10.7759/cureus.61379

**Published:** 2024-05-30

**Authors:** Jorge Rubinos Rodriguez, Santiago Fernandez, Nicholas Swartz, Austin Alonge, Fahad Bhullar, Trevor Betros, Michael Girdler, Neil Patel, Sayf Adas, Adam Cervone, Robin J Jacobs

**Affiliations:** 1 Medicine, Dr. Kiran C. Patel College of Osteopathic Medicine, Nova Southeastern University, Fort Lauderdale, USA

**Keywords:** convolutional neural networks (cnn), flow cytometry, acute lymphoblastic leukemia (all), diagnosis, classification, detection, neural networks, deep machine learning, artificial intelligence (ai)

## Abstract

Leukemia is a rare but fatal cancer of the blood. This cancer arises from abnormal bone marrow cells and requires prompt diagnosis for effective treatment and positive patient prognosis. Traditional diagnostic methods (e.g., microscopy, flow cytometry, and biopsy) pose challenges in both accuracy and time, demanding an inquisition on the development and use of deep learning (DL) models, such as convolutional neural networks (CNN), which could allow for a faster and more exact diagnosis. Using specific, objective criteria, DL might hold promise as a tool for physicians to diagnose leukemia. The purpose of this review was to report the relevant available published literature on using DL to diagnose leukemia. Using the Preferred Reporting Items for Systematic Reviews and Meta-Analyses (PRISMA) guidelines, articles published between 2010 and 2023 were searched using Embase, Ovid MEDLINE, and Web of Science, searching the terms “leukemia” AND “deep learning” or “artificial neural network” OR “neural network” AND “diagnosis” OR “detection.” After screening retrieved articles using pre-determined eligibility criteria, 20 articles were included in the final review and reported chronologically due to the nascent nature of the phenomenon. The initial studies laid the groundwork for subsequent innovations, illustrating the transition from specialized methods to more generalized approaches capitalizing on DL technologies for leukemia detection. This summary of recent DL models revealed a paradigm shift toward integrated architectures, resulting in notable enhancements in accuracy and efficiency. The continuous refinement of models and techniques, coupled with an emphasis on simplicity and efficiency, positions DL as a promising tool for leukemia detection. With the help of these neural networks, leukemia detection could be hastened, allowing for an improved long-term outlook and prognosis. Further research is warranted using real-life scenarios to confirm the suggested transformative effects DL models could have on leukemia diagnosis.

## Introduction and background

Leukemia

Leukemia, a cancer of the blood cells, results in the abnormal generation of white blood cells (WBC) in the body’s bone marrow and hinders the development of other blood components such as platelets and red blood cells. This results in a progressive, possibly fatal medical condition that requires both timely and accurate diagnosis for effective treatment and patient prognosis [1]. Leukemia is diagnosed by analyzing peripheral blood, typically under microscopy or flow cytometry. Microscopy of a blood smear allows physicians to visualize the morphological changes in blood cells associated with leukemia. However, these morphological changes are often difficult to uncover by the human eye, due to the high density of cells to sift through. Flow cytometry has a relatively fast turnaround time but requires fresh blood draws and cannot analyze the histomorphology of the blood samples [2]. Moreover, not all people with leukemia will have disease presentation in the peripheral blood. To circumvent this, an invasive bone marrow biopsy can be performed. This process has a longer turnaround time of one to two weeks but is more diagnostic, often revealing the typical hypercellular bone marrow with a drop in normal hematopoietic cells [2]. The realistic possibility of human error, the need for human input and fresh blood samples, long turnaround times, and uncomfortable procedures, raise the question of how these diagnostic processes can be streamlined to improve diagnostic rates and subsequent prognostic outcomes.

Deep learning models

Deep neural networks (DNNs), often termed deep learning (DL), are a subset of artificial neural networks inspired by the function of the human brain. DNNs are composed of interconnected artificial neurons organized into layers. Neurons in one layer are connected to neurons in the layers around it, which form a network of connections. They have revolutionized various fields, including machine learning and artificial intelligence, by enabling the development of models for tasks such as image recognition, natural language processing, and reinforcement learning [3].

Convolutional neural networks (CNNs) are specialized DNNs for completing tasks like image and video processing while recurrent neural networks (RNNs) are designed for sequential data, such as natural language text. DNNs learn from data through a process called training. During training, the network adjusts its internal parameters to minimize the difference between its predictions and the actual target values in the training data. Pre-trained DNNs have widespread medical applications since they can be fed large image datasets to produce new biomedical informatics and associations [4,5]. Yet, building these networks involves a resource-intensive and lengthy process, dependent on the quality of the input data.

Deep learning and leukemia

With the rapid advancement of DL, there has been a growing interest in leveraging these technologies for the early detection of medical conditions, which is expected to grow exponentially [6]. DNNs aid physicians in accurately diagnosing leukemia through feature analysis such as image classification, object detection, image retrieval, semantic segmentation, and human pose estimation [3,7-9]. Leukemia detection involves analyzing bone marrow smears and images to identify certain pathological features of leukemic cells while comparing them to healthy ones. Matek et al. used CNN to identify malignant WBC hematologic malignancies for one subtype of leukemia [10]. Using a dataset of 18,000 images, the system recognized the most common physiological cell type - myeloblasts - with an accuracy above 90%. Shafique et al. developed a CNN to analyze a subset of leukemia based on cell size and nucleus, finding 99% accuracy when comparing malignant cells with healthy ones [11]. Thanh et al. made a unique five-layer CNN that also found high accuracy in classifying a special subset of leukemia [12].

Researchers have used DNNs to predict the risk of leukemia development based on genetic factors. Several research groups have used these networks to predict mutations in nucleophosmin 1 (NPM1; a pathognomonic mutation for leukemia) [13-15]. Eckardt et al. developed a DL model capable of predicting NPM1 mutation status from bone marrow cytomorphology, yielding 86% accuracy [16]. Their model also completed cell segmentation and image classification to differentiate healthy cells from leukemic cells. This DL model had an accuracy of 91% when discerning a leukemia subtype cell morphology from healthy bone marrow donor samples [16].

DNNs require a large, labeled database to be trained to identify unique characteristics of the data. Ahmed et al. used data augmentation to increase the image database artificially to assist in this process [17]. Their CNN yielded an 88% accuracy for the classification of one leukemia type, and an 81% accuracy to classify classification of all leukemia subtypes [17]. Another barrier noted with the development of DNNs is the standardization of color in the images [4,18]. Saraswat et al. found a method that excludes the unwanted noise from non-standardized colors in staining called a deconvolution-based method, which performed better than simple color transfer methods [19]. This method allows the DNNs to analyze stain concentration and absorbance. Other researchers used an image pre-processing technique that adapted image color space to separate the intensity channel from hue and saturation, allowing stain concentrations and absorbance to be analyzed [20]. Another study found that when compared to non-standardized images, DNNs that used images standardized in red, blue, and green colors, had a 98% accuracy [11]. Together, these studies have found that pre-processing techniques are promising when maximizing the DNN’s ability to differentiate leukemic cells versus healthy cells.

Image pre-processing, data augmentation, and healthy versus malignant cells accuracy demonstrate the different ways researchers have used to improve DLs. The high accuracy for predicting the diagnosis of individual leukemia subtypes could open the door to an unexplored world where just one blood marrow smear/image could diagnose/differentiate from healthy cells, leukemia, and its subtypes. Also, the current procedures for leukemia detection have some drawbacks that could be alleviated by DLs, making the process more efficient, accurate, cost-effective, and reliable.

## Review

Methods

Eligibility Criteria

The inclusion for this review encompassed both experimental and nonexperimental studies, full-text articles, articles in the English language, and articles published between 2010 and 2023. The review focused on investigating DL technologies to diagnose leukemia using selected studies that used DL and its subsets, deep neural networks, rather than broader concepts such as artificial intelligence and machine learning. Abstracts, opinion pieces, presentations, and gray material were excluded.

Information Sources

**Figure 1:**
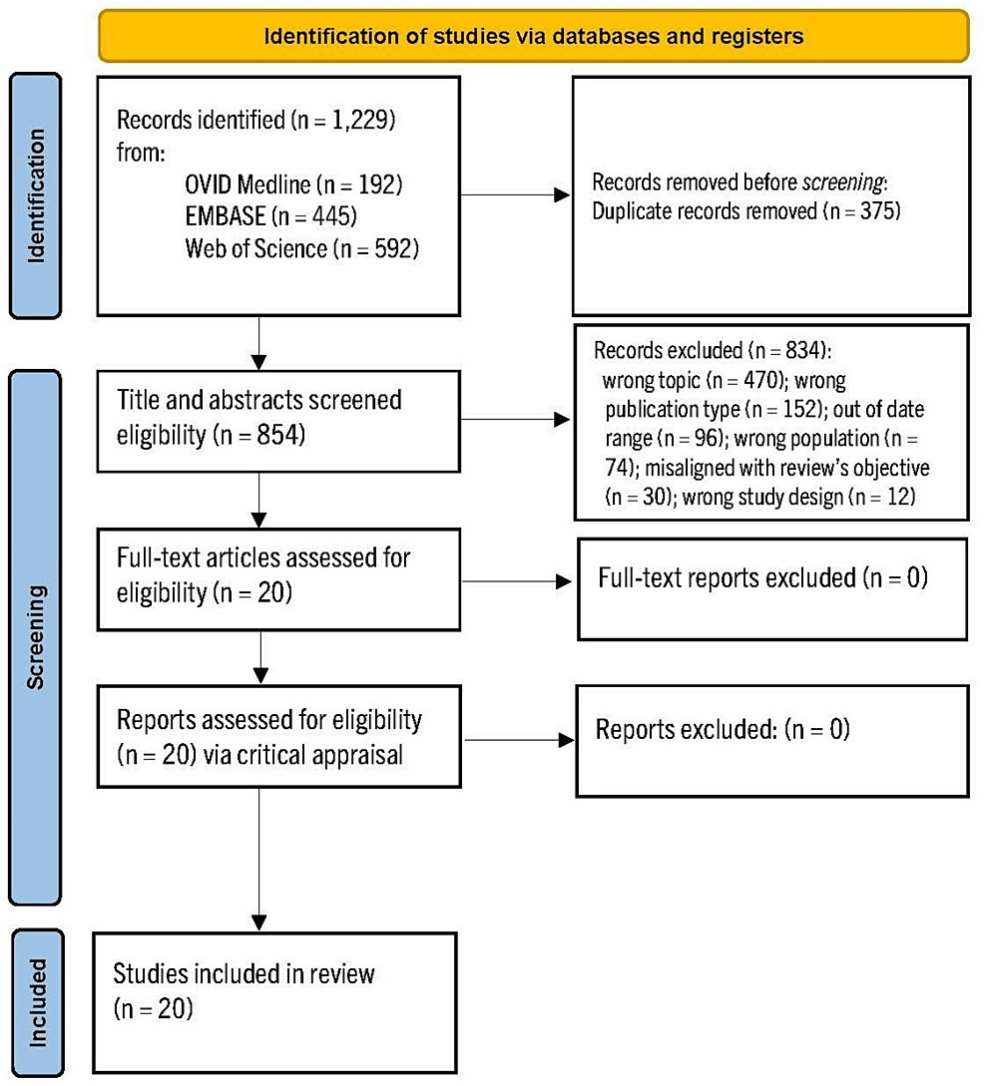
PRISMA flow diagram PRISMA: Preferred Reporting Items for Systematic Reviews and Meta-Analyses.

The search identified a total of 1,229 citations. Initially, 375 duplicates were removed, leaving 854 studies to be screened. Team members reviewed article titles and abstracts, achieving consensus about which articles warranted further consideration. Discussions continued among all three reviewers until an agreement was reached. At this point, 834 articles were excluded for not meeting screening criteria: 470 because of being the wrong topic, 152 were the wrong publication type (e.g., abstract only and dissertations), 96 based on being too old, 74 due to the wrong population, 30 did not align with the scoping review's objective, and 12 due to having the wrong study design. Consequently, 20 articles were retained for critical analysis. The screening and selection process is depicted in Figure 1.

Search Strategy

A literature search to locate published studies was conducted in September 2023. A search was performed using Embase, Ovid MEDLINE, and Web of Science. Eligible articles included those published between 2010 and 2023 and in English, utilizing the search terms “leukemia” AND “deep learning” or “artificial neural network” OR “neural network” AND “diagnosis” OR “detection,” either in the title, abstract, or keywords. A detailed search strategy table is summarized in Table 1. The reference list of all included sources of evidence was screened for additional studies. An information specialist assisted and confirmed the search strategy. Table 1 reports the search strategy.

**Table 1:** Search strategy

#	Query	Results
#1	'leukemia'/exp	437,276
#2	leuk*mia:ab,ti,kw OR leuc*mia:ab,ti,kw OR aleuk*mia:ab,ti,kw	400,219
#3	#1 OR #2	522,244
#4	'artificial neural network'/exp OR 'deep learning'/exp	117,784
#5	'deep learning':ab,ti,kw OR 'deep machine learning':ab,ti,kw OR 'neural network':ab,ti,kw	122,403
#6	#4 OR #5	160,613
#7	#3 AND #6	629
#8	detect*:ab,ti,kw OR diagnos*:ab,ti,kw OR classif*:ab,ti,kw	8,121,146
#9	#7 AND #8	439

Selection of Sources of Evidence

All identified citations were collated and uploaded into a collaborative cloud-based software application tailored for conducting systemized reviews. Members of the research team discussed the results and inclusion criteria before the initial screening of the articles generated in the primary search. Two authors then worked independently to evaluate the abstracts and titles of the publications to determine their relevance to the review. Twenty articles appeared to be relevant for the final review.

Critical Appraisal of Individual Sources of Evidence

A comprehensive evaluation of the 20 articles was performed using the critical appraisal tools developed by the Joanna Briggs Institute (JBI), known for its reliability and ongoing improvement efforts. The appropriate checklist was used for each article to consider research biases, overall coherence, and critical components contributing to article quality. Two team members independently conducted a detailed and blinded appraisal of the 20 articles chosen for the final review using the applicable JBI tools. Articles were then categorized into the high, moderate, or low risk of bias based on their scores (below 50%, between 50% and 70%, and above 70%, respectively). Articles above 70% in the criteria were included while articles under 70% were considered at higher risk for bias and thus excluded. Subsequently, the team engaged in a deliberative process to compare their appraisal scores. The relevance and quality of each article were thoroughly discussed, leading to a final consensus on selecting articles for inclusion in the review whereby all 20 articles were included in the final review.

Data Charting and Extraction Process

Two reviewers collaborated to create a data-charting form using Excel (Microsoft Corporation, Redmond, WA) and determined the data to extract. Using an iterative process, the rest of the team independently charted and engaged in discussions about the results, and continually updated the data-charting form. The information extracted was based on the article’s purpose, study population, sample, methods, limitations, and key findings (based on the percentage of success for the authors’ DL model in diagnosing leukemia and additional pertinent information).

Results

Research on the use of DL technology for leukemia detection is relatively new. The 20 articles included in this review are reported chronologically to highlight the progression of the technology used for leukemia detection over the period determined for article inclusion (2010-2023). This helps demonstrate the uniqueness of each approach to DL for leukemia detection with their percentage accuracy outcomes.

Earlier Works (2010-2018)

Two articles published in 2010-2018 fit the inclusion criteria and were thus included in the review. In 2010, Adjouadi et al. created an early version of a DL neural network model called Neural Studio, which could classify and detect leukemia with a 96.67% accuracy based on 220 blood samples (of which only 60 were abnormal) [21]. At that time, flow cytometry was still being used to create the data necessary for the neural network, so this was a new approach. Beginning in 2018 with Vogado et al., feature extraction became a staple of the more advanced convolutional neural networks (CNNs) that could be trained to do tasks. Pre-trained CNNs AlexNet, Vgg-f, and CaffeNet from a dataset of 891 images achieved over 99% accuracy in detecting features and classification using support vector machine (SVM) classifiers [22].

Years 2020-2021

Findings from seven articles published in 2020-2021 were included in this review [23-29]. Four articles published in 2020 used different models to achieve high detection accuracy [23-26] and grayscale conversion and a non-specified CNN (96.78% accuracy) [23] by (1) applying noise reduction in grayscale images with three CNN frameworks (normal group accuracy of 90% and leukemia detection accuracy of 99%) [24], (2) using a dye-sensitized solar cell (DSSCS) CNN model with novel techniques for noise suppression doing image segmentation and color normalization (reached 97.00% accuracy) [25], and (3) using color normalization only (98%) [26].

Three additional articles published in 2021 reported the start of using image augmentation and segmentation, reporting high accuracy outcomes: 99.57% accuracy with Open Neural Network Exchange (ONNX) and a You Only Look Once version 2 (YOLOv2) CNN (single-stage real-time object detection model) for feature extraction alongside an SVM for classification [27]; 98.00% accuracy using k-nearest neighbor (KNN) for feature extraction while using SVM and random forest for classification [28]; and 98.61% accuracy using fine-tuned LeukNet (the name of a new computational tool) using transfer learning for classification achieving [29].

Year 2022

Seven articles published in 2022 were included [30-36]. Anilkumar and colleagues used AlexNet (the name of a CNN architecture) and LeukNet CNNs to do dual work by feature extracting and classifying the leukemia images, attaining 94.12% accuracy [30]. Less accurate results were found using ensemble methods for feature extraction doing data augmentation, image segmentation, and red, green, and blue (RGB) to grayscale correction; the average of CNNs with SVM, K-nearest neighbor (KNN), and random under-sampling boost (RUSBoost) attaining 88.09% for leukemia detection [31].

Another article reported using ensemble and multiclass classification for classification and AlexNet, Visual Geometry Group (VGG), Residual Network-50 (ResNet-50), GoogLeNet (a type of CNN based on the inception architecture), and Dense Convolutional Network121 (DenseNet121) for feature extraction, achieving 97.04% accuracy. When conducting augmentation and image segmentation alongside DenseNet121, 97.11% accuracy was achieved [32]. Muhamad et al. obtained varying accuracy on feature extraction with softmax (amplifying effects of the exponential on any maxima in the input vector) for classification with 95.3%, 81.5%, and 97.6% on an unnamed CNN, AlexNet, and MobileNet-v2 (CNN architecture that seeks to perform well on mobile devices), respectively [33].

Other researchers applied a probabilistic neural network (PNN) for 95.705% accuracy without using data augmentation, image segmentation, or color normalization [34]. However, Sakthiraj also did not use pre-processing tools for image datasets and achieved a 99.87% accuracy with a hierarchical convolutional neural network with integrated attention and spatial optimization (HCNN-IAS) [35]. Of note, nearly 100% accuracy (average 99.7%) was attained with DarkNet-53 (backbone for the YOLOv3 object detection approach) and ShuffleNet (designed especially for mobile devices with very limited computing power) for feature extraction and SVM, ensemble methods, KNN, and naïve Bayes (an algorithm that uses Bayes' theorem to classify objects) for classification only segmenting their images [36].

Year 2023

The year 2023 demonstrated a focus on using only one model for feature extraction and classification; four articles published in this year were included in this review [37-40]. Houssein and colleagues attained 99.80% accuracy with DenseNet-161 (a model from densely connected convolutional networks) using augmentation, segmentation, and RGB (R: red, G: green, B: blue) to HSV (H: hue, S: saturation, V: value) [37], while other researchers averaged 98.15% accuracy with a CNN model using only image segmentation [38]. Naz and colleagues achieved 96.9% and 81.9% on separate datasets using AlexNet by augmenting their data and segmenting their images [39]. Wang et al., after augmenting their data, reached 92.50% accuracy with You Only Look Once eXtreme small (YOLOX-s) for feature extraction and Meta-Learning Fusion and Learning Network (MLFL-Net) for classification [40]. A summary of the articles included in this review is reported in Table 2.

**Table 2:** Summary of the articles included in the review (N = 20)

Authors	Purpose	Study samples	Methods	Limitations	Key findings
Adjouadi et al. (2010) [21]	To prove that it is possible to look at other cytometry parameters to classify blood samples into two categories (normal and abnormal) by using artificial neural networks (ANNs).	220 blood samples were considered with 60 abnormal samples and 160 normal samples.	Utilizing Beckman-Coulter flow cytometry data files containing 24 parameters, such as direct current impedance (DC), opacity (OP), and RisSoft (transformed light scanner), statistical feature extraction was employed to reduce the dataset's complexity. By computing mean, peak, standard deviation, skewness, and kurtosis from parameter histograms, a more manageable 5x93 matrix was derived for each blood sample. A binary classifier was then developed using ANNs to categorize blood samples as normal or abnormal acute lymphoblastic leukemia (ALL)/acute myeloid leukemia (AML) based on these extracted features. Receiver operating characteristics (ROC) were used to analyze its results and categorical data. Three classifiers were established with incremental data sizes (50, 100, and 130 samples), showcasing an ascending trend in sensitivity (from 80% to 96.67%) and a descending trend in the false positive rate (from 10% to 1%) as the sample size increased.	Small dataset	The AML classifier demonstrated high performance, correctly classifying 96 out of 100 normal samples and misclassifying only one AML sample as normal. The true positive (TP) fraction for AML samples was 90%, with a low false positive (FP) fraction of 2%, indicating the robustness of the classification approach for both ALL and AML cases. The results highlighted the effectiveness of the ANN-based classification system in accurately categorizing leukemia blood samples. It has been shown that increasing the size of the population does not always guarantee better results when dealing with data contaminated by high-class overlap.
Vogado et al. (2018) [22]	To test the ability of a proposed convolutional neural network (CNN) leukemia diagnosis system that does not require the segmentation process (commonly used in state-of-the-art techniques). The methodology uses pre-trained CNN models to extract features directly from the images without any previous preprocessing.	Three hybrid datasets, one with blood smears containing only one leukocyte per image, one with many leukocytes per image, and the last one with both types of images. Total images = 891.	The method proposed in this work aims to diagnose leukemia using blood smear images with a CNN using the following flow chart: feature extraction (CNN), feature selection (gain ratio), classifier (support vector machine), and image classification (pathological or non-pathological.	Small sample size	A new methodology for the diagnosis of leukemia in blood images using CNNs was reported. Based on the results obtained by the proposed approach, it was possible to validate the robustness of pre-trained CNNs for extracting features concerning classical state-of-the-art methods. Through the selection of attributes, we observed that more characteristics are required to classify the images with many leukocytes, while fewer features are required for images with only one leukocyte. The main advantage of the proposed methodology that allows it to perform better than other state-of-the-art methods is that it does not need a segmentation process.
Abou El-Seoud et al. (2020) [23]	Proposed a convolutional neural network (CNN) that detects white blood cells from microscopic images and then classifies these blood cells into one of the four classes: Class A: monocytes, Class B: lymphocytes, Class C: neutrophils, Class D: eosinophils.	Leukemic patients’ blood smears	The CNN model consisted of five layers: four layers for extracting features from input images and one output layer for classification. The dimensions of the input image were 50x50x1. The convolutional filter size is 3x3, and the max-pooling filter size is 3x3 with a stride of 1. The study emphasized the operations within the convolutional layers, including convolutional operation, activation (ReLu), and max pooling, to extract features from the input images.	The dataset excluded abnormal white blood cells, which would further help the CNN discriminate from leukemia and other diseases by only including leukemic and normal cell types.	The dataset contained images of white blood cells (neutrophils, eosinophils, monocytes, and lymphocytes). The study focused on experimenting with various hyperparameters of the CNN such as training and testing set quantities, input size, data labels, kernel size, pooling size, epochs, activation types, and the order of activations in the network. The experiments were conducted using Google Colaboratory as the notebook, Anaconda as the Python distributor, the Keras library, and TensorFlow as the backend engine. After multiple experiments, the proposed CNN model achieved an impressive accuracy rate of 96.78% in recognizing and classifying different types of white blood cells.
Huang et al. (2020) [24]	Sought to design an intelligence-assisted diagnosis method based on combining CNN and transfer learning to replace the manual interpretation of bone marrow cell morphology.	This study analyzed microscopy images of bone marrow smears from 104 subjects, including healthy individuals and patients with AML, ALL, and chronic myeloid leukemia (CML).	The researchers achieved this by constructing bone marrow cell microscopy image datasets for AML, ALL, CML, and healthy subjects and used three different CNN frameworks (GoogleNet, ResNet, and DenseNet) to construct classification models and carry out a comparative analysis. Simplified image preprocessing combined with transfer learning was used to improve the classification accuracy of the model and achieve the classification of myelograms from AML, ALL, CML, and healthy subjects.	Did not include types of leukemia with low incidence in China, such as chronic lymphocytic leukemia.	The results are fast, objective, and reliable and can avoid errors, misdiagnosis, and misjudgment due to human factors. Results showed that this method can identify subtle morphological changes that cannot be identified by the naked eye and avoid errors due to manual interpretation, which increases diagnostic accuracy. This avoids objective influencing factors caused by manual smear reading. Therefore, this method can be used to achieve standardization of bone marrow smear diagnosis.
Joshi et al. (2021) [25]	Proposed an approach to classify peripheral blood cells using a hybrid disruption-based salp swarm and cat swarm (DSSCS)-based convoluted neural network method. The hybrid approach addresses the problem of hyperparameters converging to suboptimal solutions in traditional CNNs.	This study uses a dataset consisting of 15,920 images of peripheral blood cells, which were acquired from the core Laboratory of the Barcelona, Spain clinic.	Images of peripheral blood cell smears were processed and classified into eight classes (neutrophils, basophils, eosinophils, monocytes, immature granulocytes, lymphocytes, platelets, and erythroblasts). The novel DSSCS algorithm is a combination of a modified salp swarm algorithm (SSA) and the cat swarm optimization (CSO) algorithm. The modification consists of the addition of a disruption operator to the SSA algorithm to enhance the exploration capability and diversification of the population. Once the classifications are made, the results are compared to support vector machine (SVM), neural network (NN), SVM + NN, and CNN classification methods. The performance metrics include sensitivity, specificity, and accuracy.	This study is limited to the dataset of 15,920 images provided by the clinic laboratory. A larger dataset could provide more robust results and generalizability. A more comprehensive comparison with a wider range of existing methods would strengthen the evaluation of the proposed model.	The proposed DSSCS-CNN model outperformed other existing techniques such as SVM, SVM + NN, and CNN. It achieved this by resolving the hyperparameter problem associated with the CNN architecture, leading to improved classification accuracy. The global classification accuracy was 97% delivered through the training on visual geometry group (VGG)-16 models, indicating its effectiveness in classifying peripheral blood cells. The accuracy of the proposed DSSCS-CNN model was 99%, highlighting the efficacy of the novel approach.
Kalaiselvi et al. (2020) [26]	This study aimed to improve the leukemia characteristic accuracy by scanning color and textural features from the blood image using image processing and to aid in the grouping of leukemia subtypes.	The method used nearly 10,000 microscopic blood images; however, it did not specify where the images were sourced from.	Before classification can begin, the dataset is loaded and verified to be a 200x200 red-green-blue (RGB) image. This CNN has six layers with each layer containing a convolution, activation, dropout, and max-pooling layer. A training dataset will be used to fine tune the CNN for 100 runs and an initial accuracy metric will be plotted. Then another 100 training iterations will be done to achieve sufficient accuracy. Then a test data set will be classified into four categories: ALL, CML, ALL/AML, and chronic lymphocytic leukemia (CLL).	No reports of where or when the data were collected, leading to a chance of bias.	The training accuracy consistently improves as the number of epochs increases. The accuracy achieved by the proposed architecture is 98.8%. The proposed CNN architecture achieves a validation accuracy of approximately 97%. The results demonstrate the efficacy of CNN in accurately identifying the types of leukemia in a dataset with a limited number of classes.
Amin et al. (2021) [27]	To conduct an automated approach based on deep learning that was proposed to segment and classify white blood cells (WBCs) more accurately.	6250 images, each encompassing 1250 blood smear images for five distinct WBC types.	The first step involved utilizing the WBC - Open Neural Network Exchange (ONNX) - You Only Look Once version 2 (YOLOv2) model for precise localization. Features were extracted from activation-5 LeakyReLU of the ONNX model and integrated into the YOLOv2 architecture, which comprised 26 layers in the ONNX model and nine YOLOv2 layers. To enhance classification accuracy, a Bhattacharyya rank-based feature selection approach was applied, selecting the top 500 features from a pool of 1000. These optimized features were then fed into multi-kernel SVM classifiers, including cubic SVM, quadratic SVM, O-SVM, and Gaussian SVM, for precise classification. The research utilized three benchmark datasets: acute lymphocytic leukemia image database 1 (ALL-IDB1), acute lymphocytic leukemia image database 2 (ALL-IDB2), and Leukemia Image Segmentation Challenge (LISC) dataset.	Not enough comprehensive work on leukemia image augmentation and classification.	The localization technique was validated using mean precision (mAP) and intersection over union (IoU) metrics. The results demonstrated precise localization across six types of WBCs, with the highest IoU score of 0.97 achieved for blast cells. The localization method also provided good scores for eosinophils, basophils, and lymphocytes. The second experiment used various metrics, including IoU, mean accuracy, weighted accuracy, and harmonic mean of precision and recall (F1-scores), were employed. The proposed segmentation method exhibited exceptional accuracy, with pixel-by-pixel comparisons against ground annotated images, resulting in IoU and F1 scores of 0.97 and 1.0, respectively. In the third experiment, using a multi-kernel SVM, the classification outcomes, measured in terms of accuracy, precision, recall, and F1 scores, were outstanding. The SVM with the optimized kernel achieved an overall accuracy of 98.4%.
Loddo et al. (2021) [28]	To use CNN classification to automate the analysis of digital microscopic images to identify different sub-types of WBCs and detect the presence of leukemia. This study explores the efficacy of training on cropped images and using hand-crafted descriptors.	The two datasets used are the acute lymphoblastic leukemia image database (ALL-IDB) from the Tettamanti Research Centre and the Raabin-WBC dataset.	Before training occurs, the images are preprocessed to crop individual WBCs in a bounding box (BB). This generates "tight" data, whereas uncropped images are large data. The initial step for training is the creation of hand-crafted image descriptors, consisting of moments, texture, color, and wavelet features. For the machine learning classifiers, the common K-nearest neighbor (KNN), SVM, and random forest (RF) were employed. Many CNN architectures were explored and considered using previous studies. The AlexNet, VGG-16, VGG-19, ResNet, and inception architecture were emphasized in this study. For the training step, 70% of images from the dataset were used for training and the other 30% for testing. For the validation step, this split was tweaked to 80/20 respectively. To test the robustness of the classification method, the model used to train on one dataset was tested with the images from another dataset.	This study uses the commonly used ALL-IDB dataset, limiting the scope of generalizability of the findings.	The overarching takeaway from the results is the handcrafted descriptors produced the best results when trained with the tight version of the dataset. However, the features derived from the CNNs show a trend favoring the large datasets. For example, the average accuracy for the CNN trained on a large dataset for WBC is 97.9% versus the range of 88.9% for the best machine learning classifier RF. For the ALL-IBD dataset, the best machine learning performer was the KNN model with an average accuracy percentage of 65.2%, similar to 67.6% for the CNN. A similar story can be told for the models trained on tight datasets. CNNs trained on the tight data produced impressive results when tested on tight images, with performance exceeding 90%. There was a general decline in accuracy across the board when models trained on large datasets were tested with tight images, and vice versa.
Vogado et al. (2021) [29]	To evaluate a CNN generalization ability to diagnose leukemia from images of different resolution, contrast, color, and texture characteristics using a cross-dataset validation technique.	LeukNet, a model of convolutional neural network for the diagnosis of leukemia was evaluated on 3536 images of blood smears belonging to different sources, including hospitals and other institutions. Each dataset includes images acquired under different conditions, dimensions, and characteristics of color, contrast, and texture.	The experimental protocol used a leave-one-dataset-out cross-validation where the test is carried out in one dataset and the remaining datasets are used in the training process. This procedure is performed until all datasets are tested individually. This ensures that the CNN is not trained with any image of the datasets to be tested	Small sample size	From the comparisons performed against previous studies, some conclusions may be drawn as to the computational leukemia diagnosis from images. First, fine-tuning may be more efficient than off-the-shelf feature extraction. Second, CNNs with more representations through feature maps perform better in cross-dataset experiments. Furthermore, the choice of the fine-tuning technique is essential for the correct definition of CNN parameters. As for blood sample images belonging to a different domain than those used to pre-train the layers, adjusting all the layers is preferable.
Anilkumar et al. (2022) [30]	To classify ALL into B-cell ALL and T-cell ALL according to the WHO scheme, using deep learning (DL) techniques applied to a publicly available dataset, without relying on traditional image segmentation or hand-crafted feature extraction methods, thereby avoiding intensive computational processes.	56 peripheral blood smear images, containing both B-cell and T-cell ALL samples, 168 individual lymphoblast images representing B-cell ALL, and another 168 lymphoblast images representing T-cell ALL from these original images.	The method for the classification of ALL blood smear images into B-cell ALL and T-cell ALL was conducted using two deep CNNs: AlexNet, a pre-trained deep CNN, and LeukNet, a custom-designed CNN. Image segmentation and hand-crafted feature extraction were avoided. The images were cropped to fit the input data size of the CNNs (227 × 227 for AlexNet and 224 × 224 for LeukNet). Data augmentation techniques such as flipping, rotation, translation, and scaling were applied to avoid overfitting due to the limited number of images. Transfer learning was employed for AlexNet, where the last three layers were adjusted to match the number of classes. The LeukNet architecture comprised 17 layers, including convolution, Rectified Linear Unit (ReLU) activation, cross-channel normalization, max pooling, fully connected, dropout, and softmax layers, with a depth of 5.	Small dataset	For AlexNet, a classification accuracy of 94.12% was achieved with adaptive moment estimation (ADAM), with a validation accuracy of 93.94% and a training time of 8 minutes and 27 seconds. Similar results were obtained with RMSprop. LeukNet, with a smaller depth, achieved a classification accuracy of 94.12% using all three algorithms, with the fastest training time of 2 minutes and 26 seconds using stochastic gradient descent with momentum (SGDM). Sensitivity and specificity were comparable between AlexNet and LeukNet with ADAM and root mean square propagation (RMSprop), while LeukNet outperformed AlexNet with SGDM in detecting T-cell ALL. The study highlighted the simplicity of their classification framework, emphasizing the absence of complex image segmentation and feature extraction methods.
Baig et al. (2022) [31]	To introduce a computer-aided method utilizing deep learning techniques to detect blood cancer through microscopic images.	4150 images of blood smears.	The methodology followed in this study starts first by having pre-processing applied to enhance image clarity, followed by image segmentation to extract the area of interest. Hybrid CNN models, namely, CNN-1 and CNN-2, are employed for feature extraction from training images. These features are fused using canonical correlation analysis (CCA) to create discriminative vectors. The study emphasizes the importance of pre-processing, including RGB to greyscale conversion, image adjustment, adaptive histogram equalization, and noise removal. Data augmentation techniques are used to address limited data availability and potential overfitting issues. The classification stage involves various classifiers such as bagging ensemble, linear programming boost (LPBoost), total boost ensemble, KNN, fine K-nearest neighbors (FK-NN), RUSBoost, coarse KNN, and SVM, aimed at predicting cancer types based on selected features. Transfer learning is applied to reduce the complexity of training CNN models.	None noted	Two parallel CNN models, CNN-1 and CNN-2, were trained for feature extraction, achieving individual class accuracies of 77.27% ALL, 98.91% AML, and 92.22% multiple myeloma (MM) for both models. A Canonical correlation analysis (CCA) fusion technique was employed to concatenate the extracted features, which were then classified using various traditional machine learning algorithms, including bagging ensemble, total boost, FK-NN, RUSBoost, coarse KNN, SVM, LPBoost, and active contours. The Bagging ensemble model with CCA fusion exhibited the highest accuracy at 97.04%, outperforming other classifiers.
Claro et al. (2022) [32]	To assess how employing data augmentation and combinations of CNNs impacts the identification of various types of leukemia in blood slide images.	3,536 images, 1,434 images belong to the healthy blood slide (HBS) class (40.55% of the total), 881 belong to the ALL class (24.92%), 978 belong to the AML class (27.66%) and 243 belong to the ‘‘other types” class (6.87%).	Techniques of data augmentation were applied to increase the generalization capacity of the classification models. The study used 18 public datasets, ensuring that the datasets had ground-truth information and were previously used (as reported in published literature). Data augmentation techniques, such as rotation, translation, flipping, scaling, and shear transformations, were applied to tackle the limited availability of training data and class imbalances. Various pre-trained CNN architectures, including AlexNet, VGG-16 and 19 (VGG16 and VGG19), ResNet (ResNet50), GoogLeNet (InceptionV3 and Xception), and DenseNet121, were evaluated. Two configurations were explored: a multilevel configuration, where feature maps from different CNNs were concatenated and fed into a fully connected layer, and an ensemble technique, where predictions from multiple CNNs were combined using majority voting. To assess the classification results, metrics such as accuracy, precision, recall, F1-score, and the kappa index were computed.	Not enough comprehensive work on leukemia image augmentation and classification.	Using K-fold cross-validation (a technique for evaluating predictive models), the impact of data augmentation (DA) on binary classifications was assessed: leukemia vs. healthy slides, ALL vs. healthy slides, and AML vs. healthy slides. In the first scenario, ResNet50-DA excelled, benefiting significantly from DA. In the ALL vs. healthy slides scenario, DenseNet121-DA exhibited superior accuracy, recall, F1-score, and kappa index, while ResNet50 excelled in precision. AML vs. healthy slides favored ResNet50-DA for accuracy, precision, F1-score, and kappa index, with DenseNet121 showcasing high recall. Multiclass classifications, particularly ALL vs. AML vs. healthy slides, displayed a slightly reduced performance overall, but DenseNet121-DA still achieved a notable accuracy of 97.11%. In a more complex multiclass scenario, including other leukemia types, DenseNet121 outperformed others with values above 94%, excelling in accuracy, precision, recall, and F1-score, with rotation as the most effective DA technique. Ensemble models, particularly DenseNet121-DA, emerged as the most effective approach.
Muhamad et al. (2022) [33]	To use different CNN classification models to detect and differentiate white blood cells into basophils, eosinophils, lymphocytes, monocytes, and neutrophils.	The patient data was sourced from the Hiwa Cancer Hospital in Sulaymaniyah-Iraq. A total of 1728 images were used.	Transfer learning was used by employing pre-trained neural networks, such as CNN, CNN MobileNetv2, and CNN Alex Net, to process their collected images. The collected images were analyzed using the feature extractor of previous models to finetune the classification. This method was used to reduce the amount of data processing required to generate an effective model, and therefore simplify the training procedure. An 80/20 split of images was set for training and testing, respectively.	Using previously trained models can lead to bias in results depending on which datasets were used to train them. The patient data set from one specific hospital limits the generalizability of the findings.	The fine-tuned models yielded an accuracy of 95.2% for the CNN model, 97.2% for the MobileNet2 model, and 81.5% for the AlexNet model. However, the researchers note that despite different designs with different numbers of layers, parameters, or branches, the overall performance of the models doesn't differ much.
Prabhakar et al. (2022) [34]	To improve the use of microarray techniques using probabilistic neural networks to classify leukemia, specifically ALL and AML.	The dataset referenced by Golub. The dataset contained 7129 genes, of which 47 samples of ALL and 25 samples of AML were found.	Two-level feature selection employing statistical tests was first used for best gene selection. Minimum redundancy maximum relevance (MRMR), signal-to-noise ratio (SNR), multivariate error weight uncorrelated shrunken centroid (EWUSC), and multivariate correlation-based feature selection (CFS) were chosen as the initial feature selection techniques. This output was then optimized through 5 different optimization techniques (African Buffalo optimization (ABO), Artificial Bee Colony Optimization (ABCO), Cockroach Swarm Optimization (CSO), Imperialist Competitive Optimization (ICO), and Social Spider Optimization (SSO). Lastly, all these optimized values were fed for example, MRMR with SSO to varying classifiers including Naive Bayes Classifiers (NBC), SVM, RF, and a PNN for the classification of AML and ALL. Specificity, sensitivity, and accuracy performance indexes were included in the study.	Mentions the "curse of dimensionality" - that the number of variables at the genetic level far exceeds the number of samples.	A probabilistic neural network (PNN) classifier with 200 genes at multivariate correlation-based feature selection (CFS) attained the highest accuracy of 95.705%. The Performance Index (PI) parameter for four classifiers averaged in five different optimization methods listed in the methods. NBC classifier with 200 genes selection for the ABO algorithm peaked with the highest PI of 74.33%. The study breaks down the average performance of each classifier with a respective initial feature selection technique.
Sakthiraj (2022) [35]	To introduce an integrated framework that combines advanced data augmentation, deep learning architectures, and optimization techniques to significantly improve the accuracy, reliability, and efficiency of leukemia classification and detection, while integrating alongside something they call the Internet of Medical Things (IoMT).	American Society of Hematology (ASH) image databank	The IoMT framework integrates internet of things-enabled medical sensors and electronic health records (EHR), allowing patients to acquire and transmit their medical data, including leukemia-related information, to a hybrid CNN-IAS (Interactive Autodidactic School Optimization) model for diagnosis. The proposed hybrid model is detailed, starting with data augmentation techniques to enhance the leukemia dataset, followed by effective classification of leukemia subsets such as healthy, CML, CLL, AML, and ALL. The IoMT framework enables home-based treatment, facilitating real-time disease monitoring and diagnosis, thereby minimizing costs. The hybrid CNN-IAS (a lightweight convolutional neural network) algorithm performs feature extraction, fusion, and classification. The proposed model's performance is evaluated using precision, recall, accuracy, and F1 score metrics, achieving remarkable accuracy rates for different leukemia subtypes.	Relied solely on a single database, without specifying the quality or quantity of the data it contained.	The IoMT-based hierarchical CNN with integrated attention and spatial optimization (HCNN-IASO) model achieved an exceptional accuracy of 99.87%, as evidenced by a comprehensive performance analysis, including metrics such as precision, recall, accuracy, and F1 (a measure of predictive performance) score. Comparisons with state-of-the-art techniques highlighted the superiority of the proposed HCNN-IASO model. The study also emphasized the importance of subtype detection for precise therapy and risk reduction, showcasing the potential of the IoMT-based approach in advancing the field of leukemia diagnosis. Furthermore, HCNN extracted features from the leukemia dataset that were data augmented, and it proved paramount to add an attention layer to fuse these features. Another layer “SoftMax” functioned as a classifier, categorizing the leukemia dataset into various subtypes. To further optimize the classification accuracy, IASO techniques were used.
Saleem et al. (2022) [36]	The objective of this study is to introduce a modified deep learning methodology designed to achieve precise segmentation of leukocytes and their classification.	ALL-IDB1 and two databases with 107 and 260 images, respectively; LISC dataset consisting of hematological images collected from 400 samples divided into lymphocytes, monocytes, neutrophils, eosinophils, and basophils. Note: The ALL-IDB1 can be used both for testing the segmentation capability of algorithms, as well as the classification systems and image preprocessing methods. The images are taken with different magnifications of the microscope ranging from 300 to 500.	The methods begin in experiment 1, where classification is performed using a fusion of deep models. The classified images are then passed to experiment 2, where required regions are segmented using two different approaches: a statistical segmentation method based on color-based morphological thresholding and a deep semantic neural network. To augment the datasets, a generative adversarial network model was used, increasing the dataset sizes significantly. Feature extraction was carried out using pre-trained deep CNN models, DarkNet-53, and ShuffleNet. The feature selection process involves using principal component analysis to reduce dimensionality. Selected features from DarkNet-53 and ShuffleNet are then fused to create a final feature vector. For leukemia classification and white blood cell type recognition, various machine learning algorithms such as SVM, KNN, ensemble methods, decision trees, and naïve Bayes are employed. Two segmentation techniques are used: a statistical morphological approach based on color conversion and morphological operations, and a deep semantic segmentation model utilizing Deeplab V3+ and ResNet-18 networks.	Excess segmentation could ultimately lead to the omission of key data and decrease the accuracy.	In experiment 1, the authors used two datasets, LISC and ALL-IDB, and implemented a classification task using various classifiers. The classification results reached 100.0% accuracy for ALL-IDB and 99.70% for LISC datasets. The ensemble subspace KNN classifier achieved the highest accuracy accounting for 100% on the ALL-IDB database. The researchers also compared their approach to existing algorithms and showed superior results. In experiment 2, the authors applied segmentation techniques to the classified LISC dataset. They used both statistical morphological-based segmentation and semantic segmentation using deep learning models like Darknet-53 and ShufeNet. The segmentation results were impressive, with an average accuracy of over 90% for most cell types. The proposed DeepLab V3+ and ResNet-18-based semantic segmentation achieved a global accuracy of 98.6% and outperformed existing methods. The statistical morphological-based segmentation achieves an average accuracy of 85.95% (except for lymphocytes).
Houssein et al. (2023) [37]	The purpose of this study is to present an improved, lightweight, and effective computer-aided diagnosis (CAD) system that can automatically classify four types of leukocytes (neutrophils, eosinophils, lymphocytes, and monocytes), which is a significant contribution to the field of medical image analysis. The author investigated the potential of DenseNet-161 pre-trained CNN for the suggested CAD system, which is a modern approach to developing the system.	The Blood Cell Count and Detection (BCCD) database was split into two sets: approximately 80% of the data (9,966 images) for the training set and 20% (2,487 images) for the validation set. The training set was composed of 2,497, 2,483, 2,487, and 2,499 images, while the validation set contains 623, 620, 620, and 624 images of eosinophil, lymphocyte, monocyte, and neutrophil.	Images of blood cells in a microscopic smear were collected from GitHub, a public source that uses the MIT license. An end-to-end CAD system for leukocytes has been created and implemented as part of this study. The introduced system comprises image preprocessing and enhancement, image segmentation, feature extraction and selection, and WBC classification. By combining the DenseNet-161 and the cyclical learning rate (CLR), the authors contributed an approach that speeds up hyperparameter optimization. They also offered the one-cycle technique to rapidly optimize all hyperparameters of DL models to boost training performance.	Accuracy, precision, and recall were presented as indicators of the suggested model’s efficacy. The authors claimed to have solved the multiclass classification problem with a raw data accuracy (ACC) of 99.8%.	Using a combination of the recently developed pre-trained CNN, DenseNet, and the one-fit cycle policy, this study describes a training technique for the classification of white blood cells for leukemia detection. The proposed method is more accurate compared to the state of the art.
Kadmin et al. (2023) [38]	This study proposed the use of a CNN classifier to detect acute myeloid leukemia from a single blood smear.	The ALL-ADB1 database is provided by the American Society of Hematology. It contains 100 photos, with 45 showing blast cells and the remaining 55 showing non-blast cells. There are an estimated 35,000 distinct blood components in these images. The lymphocytes in this dataset have been classified by oncology specialists.	Images of a single-cell blood smear dataset are characterized into four classes. The classes are benign, early, pre, and pro. These images are fed into a CNN consisting of filtering layers to extract features from the segmented image samples. The combination of convolutional, pooling, and fully connected layers creates a deep convoluted neural network. Weight parameters are fed into each layer to perform the output compilation. Segmented cells' features include shape, color, and texture properties. The images are processed through a CNN which evaluates specific cell features to classify them into four categories. Each category metric is based on accuracy, precision, recall, and an F1 score.	This study uses a relatively small dataset of only 100 images. Also, performance metrics are not compared to established methods of processing images with machine learning.	The accuracy percentages for the benign, early, pre, and pro categories are 96%, 97%, 99%, and 99%, respectively. The system provided can perform various automated processing tasks including color correlation, segmentation of nucleated cells, and efficient validation and categorization. The results indicate that this characteristic effectively distinguishes between cancerous and normal cells with reliability.
Naz et al. (2023) [39]	The purpose of this study was to develop an automated, robust, and efficient classification and detection system for leukocytes in microscopic blood images.	The Local Initiatives Support Corporation (LISC) database has 400 microscopic blood images turned into 3,600 samples and is sourced from the Australian National Database. The Dhruv set was said to be increased to 10,000 samples from the Dhruv set. The origin of the Dhruv set is not stated.	The images are preprocessed and augmented so that the cells are isolated, generating many cell samples per image. Then a wavelet transformation is applied to the augmented data to extract low- and high-frequency information. CNN training is based on this frequency information. The leukocytes are classified into five main types: eosinophils, basophils, neutrophils, monocytes, and lymphocytes. The proposed method is compared to the existing models of Jianweib, Seyed, and hierarchical SVM.	The article did not mention where the Dhruv dataset was collected from, leading to a bias potential.	The proposed model yielded an accuracy of 96.9% for the LISC dataset and 81.9% for Dhruv's dataset.
Wang et al. (2023) [40]	Explore the efficacy of an artificial intelligence-assisted diagnosis support system of morphological examination based on bone marrow smears including cell detection, classification, and prediction of leukemia types.	A large-scale dataset of 11,788 fully annotated micrographs from 728 smears and 131,300 expert-annotated single-cell images.	Bone marrow smears were processed by the Wright Stain and digitized with the microscope (Olympus BX50, Tokyo, Japan) and the camera (JEDA SmartV 650D). First, the entire smear was captured by experts under a 10x objective lens in the view of the microscope to select the region of interest. Second, 10 to 20 micrographs for each smear were captured under a 10x eyepiece and a 100x oil immersion objective. The numerical aperture of the oil immersion objective was 1.3 and the dimensions of the micrographs were 1920 × 2560. Finally, with the labeling software developed, experts localized cells in the micrographs and determined the ground truth category label for every single cell. For a single smear, they obtained the final diagnostic opinion and 200 to 300 valid cells with bounding boxes by routine practice.	Only used one dataset	For diagnosing types of acute leukemia, some types achieved 100% accuracy, including acute lymphoblastic leukemia/lymphoma and acute monoblastic/monocytic leukemia. Meanwhile, some patients with acute myelomonocytic leukemia were predicted to have acute myeloid leukemia with maturation. This is due to lower monoblasts classification performance, where many monoblasts were classified into myeloblasts, like expert human hematopathologists. The overall total accuracy was 92.5%, reflecting the potential for assisted diagnosis.

Table 3 reports the deep learning techniques and tools and accuracy attained in detecting leukemia used in each study included in this review.

**Table 3:** Deep learning techniques and accuracy attainment for leukemia detection

Year	Author	Image dataset augmentation	Segmentation	Color normalization	Neural network - feature extraction	Neural network - cell classification	Accuracy
2010	Adjouadi et al. [21]	No	No	No	Not applicable	Neural Studio - artificial neural network	96.67%
2018	Vogado et al. [22]	No	No	No	AlexNet, Visual Geometry Group fast (Vgg-f), and CaffeNet	Support vector machine classifier	>99%
2020	Abou El-Seoud et al. [23]	No	No	Yes, grayscale version	Not applicable	Not specified	96.78% differentiating between 5 white blood cell types
	Huang et al. [24]	No	No	Yes, "noise reduction"	Three different convolutional neural network (CNN) frameworks (GoogleNet, ResNet, and DenseNet), fine-tuning seen (2018 connection)		Normal group: 90%; leukemia average: 97%
	Joshi et al. [25]	No	Yes	Yes, plus "noise suppressant"	The disruption-based salp swarm and cat swarm convolutional neural network (DSSCSCNN) method	The DSSCSCNN method	97%
	Kalaiselv et al. [26]	No	no	Yes	Not applicable	Not applicable	98%
2021	Amin et al. [27]	Yes	Yes	Yes "noise removal"	Open Neural Network Exchange (ONNX) a You Only Look Once version 2 (YOLOv2 model)	Support vector machine (SVM) classifier	99.57%
	Loddo et al. [28]	Yes	Yes	no	The common K-nearest neighbor (KNN)	Support vector machine (SVM), and random forest (RF)	98%
	Vogado et al. [29]	Yes	Yes	No	LeukNet (Fine tuning VCG-16)	Transfer learning	98.61%
2022	Anilkumar et al. [30]	Yes	No	Yes	AlexNet, a pre-trained deep CNN, and LeukNet, a custom-designed convolutional neural network (CNN)	AlexNet, a pre-trained deep CNN, and LeukNet, a custom-designed CNN	94.12%
	Baig et al. [31]	Yes	Yes	Yes, red-green-blue (RGB) to grayscale	Convolutional neural network (CNN) 1 and 2 (CNN-1, CNN-2)	Support vector machine (SVM), bagging ensemble, total boosts, RUSBoost, and fine KNN	Bagging ensemble: 97.04%; leukemia average: 88.09%
	Claro et al. [32]	Yes	Yes	No	AlexNet, Visual Geometry Group (VGG), ResNet-50, GoogLeNet, DenseNet121	Ensemble and multiclass classification	DenseNet121: 97.11%; multiclass: 94%
	Muhamad et al. [33]	Yes	Yes	No	Convolutional neural network (CNN), AlexNet, and MobileNet-v2	SoftMax	CNN: 95.3%; AlexNet: 81.5%; MobileNet-v2: 97.6%
	Prabhakar et al. [34]	No	No	No	Probabilistic neural network (PNN)	Minimum redundancy maximum relevance (MRMR), signal-to-noise ratio (SNR), error weight uncorrelated shrunken centroid (EWUSC), and correlation-based feature selection (CFS)	PNN: 95.705%
	Sakthiraj et al. [35]	No	No	No	Hybrid convolutional neural network with invasive alien species (HCNN-IAS)	HCNN-IAS	99.87%
	Saleem et al. [36]	No	Yes	Yes, red-green-blue (RGB) in hue saturation value (HSV)	DarkNet-53 and ShuffleNet	Support vector machine (SVM), K-nearest neighbors (KNN), ensemble methods, decision trees, and naïve Bayes	KNN, ensemble: 99.7%; DarkNet-53 and ShuffleNet: 90% for all subtypes
2023	Houssein et al. [37]	Yes	Yes	Yes, red-green-blue (RGB) in hue saturation value (HSV)	DenseNet-161 model with single-cycle policy	DenseNet-161 model	99.80%
	Kadmin et al. [38]	No	Yes	No	CNN model	CNN model	Average: 98.15%
	Naz et al. [39]	Yes	Yes	No	AlexNet	AlexNet	96.9% and 81.9% on separate datasets
	Wang et al. [40]	Yes	No	No	YOLOX-s model	MLFL-Net	92.50%

Discussion

This review explored the development of CNN, a type of deep learning architecture commonly used in computer vision, image analysis, and other spatial data processing tasks, in leukemia detection from 2010 to 2023. Selected papers are depicted chronologically and their various methods with accuracy outcomes and current limitations are reported.

Implication of Early DL Models for Leukemia Detection

Early works, like Adjouadi and colleagues in 2010, laid a foundation even if they lacked the resources of their successors, achieving a notable 96.67% accuracy in leukemia cell classification using Neural Studio (an early neural network model) [22]. During this period, however, the methods heavily relied on flow cytometry for data (bone marrow blood samples) acquisition, a technique not seen in more recent studies due to the ability to train networks for feature extraction [21]. This can be seen in 2018 when researchers utilized several pre-trained CNN models (AlexNet, Vgg-f, and CaffeNet) for feature extraction and an SVM cell classifier [29], achieving an accuracy score of 99%, which set the new standard [21,22].

These early findings demonstrated that the trained models were nearly perfect in feature extraction but had to rely on SVM to do cell classification [22]. These works provide a historical context for the evolution of neural network-based methods in leukemia cell classification. It also highlights the early stages of using CNN and how these early methods were a foundation for later advancements. Moreover, by noting the accuracy rates of these early works and comparing them to subsequent ones, the accuracy of leukemia cell classification has improved over time (i.e., 96.67% accuracy in 2010 and 99% accuracy in 2018). The shift from earlier methods like Neural Studio in 2010 used flow cytometry data from bone marrow blood samples, while later methods, like those from 2018, used pre-trained CNNs and SVMs, indicating technological advancements and different approaches in extracting and classifying features. This methodological shift is important as it demonstrates how technology has moved from specialized techniques to more generalized approaches that leverage advances in DL. Providing these evolutionary insights into how the use of CNNs and related technologies has advanced can help guide future research directions and clinical applications.

Implication of More Recent DL Models

From 2020 to 2022, innovations like grayscale conversion, noise reduction, DSSCS CNN model, and color normalization yielded excellent results in the accuracy of CNN, with percentages ranging from 81.5% to 99.57% [23-27]. The DSSCS CNN model was distinct from the others as it handled feature extraction and cell classification, a departure from previous approaches that used separate techniques like SVM, bagging, and multiclass ensembles [29]. This model represents a more integrated approach, likely leading to more efficient processing and potentially better accuracy. The accuracy ranges from 81.5% to 99.57%, showing the significant strides made during this time. It demonstrates that these innovations contributed to more reliable and accurate classification. Furthermore, during this time, the introduction of image augmentation and segmentation, signaling a growing emphasis on improving their own CNN models through training with larger artificial datasets and advanced feature extraction methods, was explored. Image augmentation has proven especially helpful by allowing datasets to be augmented from only a few images, increasing the dataset pool without needing more bone marrow samples, which is useful where large patient datasets are difficult to find. This could contribute to more efficient and cost-effective research and development of neural network models [28-36].

Implication of the Paradigm Shift With Current DL Models

In 2023, an important shift occurred from using several CNNs for specific tasks to applying a single model for feature extraction and cell classification, with DenseNet-161 and AlexNet achieving 99.80% and 96.90%/81.90%, accuracy, respectively [37-40]. However, the highly variable accuracy rates demonstrate the need for more research and replication of these studies.

This shift represents a transition toward simplicity and efficiency in model architecture, reducing the complexity of the pipeline. The use of DenseNet-161 shows that DL models with densely connected layers can be highly effective for feature extraction and classification. Its architecture enhances the model's ability to learn complex features without excessively increasing model size. This approach can be efficient, improve model performance, and make it more feasible to implement in clinical settings.

The Potential of DL Technology for Leukemia Detection

The strides made in DL for leukemia detection in the 13 years of published evidence covered in this review (2010-2023) demonstrate the potential of this technology to assist in the more efficient detection of leukemia. Integrating image augmentation, segmentation, and more advanced CNN architectures shows promise. The range of innovative methodologies suggests an ongoing need to refine and enhance performance. The various unique approaches suggest an ongoing effort to streamline processes and optimize performance. However, these models have not been validated in real-world clinical settings, where patient outcomes rely heavily on the diagnostic accuracy of the models.

Despite promising accuracy rates, challenges such as dataset variability, model interpretability, and generalization to diverse patient populations persist. This limits the validity of comparisons between studies since the different datasets and tools affect the accuracy scores. Excess segmentation and augmentation can lead to artifacts and omission of key data, thereby decreasing the validity of its reported accuracy [36]. Also, images contain immense amounts of genetic variables that artificial enhancement cannot replicate through augmentation. While it is good for training the CNN models to detect several variables, the genetic variables that need to be examined vastly outnumber the number of valid samples currently found, something the researchers called the “curse of dimensionality” [34]. Future research should prioritize addressing these challenges before it can be used in a real patient setting. Datasets need to be improved and conducting validation studies in clinical settings will prove their actual beneficial factor. Therefore, collaborative efforts among researchers on a global scale are essential to tackle these limitations.

Limitations

While the methods used in conducting the scoping review used rigorous and transparent methods throughout the process, some limitations exist. This review may not have been able to identify all the articles in the published literature despite attempts to be as thorough as possible. The search phrase used included several different words and phrases used in the literature to describe deep learning and leukemia detection, but other terms may also exist. Moreover, the search included three major medically focused databases, but searching other online databases may have produced additional articles. Also, we selected articles that were only in English, so including articles published in other languages might have yielded more studies. The findings from this review should be approached with a critical awareness of these limitations and recognize their potential impact on the comprehensiveness, generalizability, and relevance of the synthesized evidence. The limitations of each article in the final review are reported in Table 2.

Implications for future research

Based on the results of this review, future research in leukemia detection using deep learning models could focus on enhancing the accuracy, efficiency, and applicability of the models discussed. Exploring new models to augment existing datasets should consider differences in patient demographics, disease subtypes, and data acquisition techniques (e.g., gene expression profiles and diverse imaging modalities). Future research could focus on developing ways to improve the robustness of models, addressing variations in data quality, acquisition protocols, and patient populations to mitigate risks of overfitting. This would serve to improve generalization performance. A salient gap in DL research involving leukemia detection appears to be in the clinical validation of DL models for leukemia detection. While DL has shown promising results in research settings, their performance in real-world clinical environments may vary.

## Conclusions

This review presents a comprehensive overview of the evolution of CNNs in leukemia detection from 2010 to 2023, highlighting significant advancements and emerging trends in the field. The initial studies laid the groundwork for subsequent innovations, illustrating the transition from specialized methods to more generalized approaches capitalizing on DL technologies for leukemia detection. This summary of recent DL models revealed a paradigm shift toward integrated architectures, resulting in notable enhancements in accuracy and efficiency. Regardless, impediments (e.g., real-world clinical setting validation, variability in datasets, and model interpretability) remain substantial barriers to widespread adoption. While DL technology holds promise for transforming leukemia detection, existing limitations must be overcome via rigorous research and validation practices. Future initiatives should enhance model accuracy, efficiency, and clinical applicability to fully leverage DL technology in improving leukemia diagnosis and patient outcomes.

## References

[1] Rezayi S, Mohammadzadeh N, Bouraghi H, Saeedi S, Mohammadpour A (2021). Timely diagnosis of acute lymphoblastic leukemia using artificial intelligence-oriented deep learning methods. Comput Intell Neurosci.

[2] Blackburn LM, Bender S, Brown S (2019). Acute leukemia: diagnosis and treatment. Semin Oncol Nurs.

[3] Guo Y, Liu Y, Oerlemans A, Lao S, Wu S, Lew MS (2016). Deep learning for visual understanding: a review. Neurocomputing.

[4] Niazi MKK, Parwani AV, Gurcan MN (2019). Digital pathology and artificial intelligence. Lancet Oncol.

[5] Tizhoosh HR, Pantanowitz L (2018). Artificial intelligence and digital pathology: challenges and opportunities. J Pathol Inform.

[6] Shah NR (2019). Health care in 2030: will artificial intelligence replace physicians?. Ann Intern Med.

[7] Lhermitte L, Mejstrikova E, van der Sluijs-Gelling AJ (2018). Automated database-guided expert-supervised orientation for immunophenotypic diagnosis and classification of acute leukemia. Leukemia.

[8] Wang F, Casalino LP, Khullar D (2019). Deep learning in medicine-promise, progress, and challenges. JAMA Intern Med.

[9] Rawat W, Wang Z (2017). Deep convolutional neural networks for image classification: a comprehensive review. Neural Comput.

[10] Matek C, Schwarz S, Spiekermann K, Marr C (2019). Human-level recognition of blast cells in acute myeloid leukaemia with convolutional neural networks. Nat Mach Intell.

[11] Shafique S, Tehsin S (2018). Acute lymphoblastic leukemia detection and classification of its subtypes using pretrained deep convolutional neural networks. Technol Cancer Res Treat.

[12] Thanh TTP, Vununu C, Atoev S, Lee SH, Kwon KR (2018). Leukemia blood cell image classification using convolutional neural network. Int J Comput Theory Eng.

[13] Rose D, Haferlach T, Schnittger S, Perglerová K, Kern W, Haferlach C (2014). Specific patterns of molecular mutations determine the morphologic differentiation stages in acute myeloid leukemia (AML). Blood.

[14] Falini B, Bolli N, Liso A, Martelli MP, Mannucci R, Pileri S, Nicoletti I (2009). Altered nucleophosmin transport in acute myeloid leukaemia with mutated NPM1: molecular basis and clinical implications. Leukemia.

[15] Park BG, Chi HS, Jang S (2013). Association of cup-like nuclei in blasts with FLT3 and NPM1 mutations in acute myeloid leukemia. Ann Hematol.

[16] Eckardt JN, Middeke JM, Riechert S (2022). Deep learning detects acute myeloid leukemia and predicts NPM1 mutation status from bone marrow smears. Leukemia.

[17] Ahmed N, Yigit A, Isik Z, Alpkocak A (2019). Identification of leukemia subtypes from microscopic images using convolutional neural network. Diagnostics (Basel).

[18] Rehman A, Abbas N, Saba T, Rahman SI, Mehmood Z, Kolivand H (2018). Classification of acute lymphoblastic leukemia using deep learning. Microsc Res Tech.

[19] Saraswat M, Arya KV (2014). Automated microscopic image analysis for leukocytes identification: a survey. Micron.

[20] MoradiAmin M, Memari A, Samadzadehaghdam N, Kermani S, Talebi A (2016). Computer aided detection and classification of acute lymphoblastic leukemia cell subtypes based on microscopic image analysis. Microsc Res Tech.

[21] Adjouadi M, Ayala M, Cabrerizo M, Zong N, Lizarraga G, Rossman M (2010). Classification of leukemia blood samples using neural networks. Ann Biomed Eng.

[22] Vogado LHS, Veras RMS, Araujo FHD, Silva RRV, Aires KRT (2018). Leukemia diagnosis in blood slides using transfer learning in CNNs and SVM for classification. Eng Appl Artif Intell.

[23] Abou El-Seoud S, Siala MH, McKee G (2020). Detection and classification of white blood cells through deep learning techniques. Int J Online Biomed Eng.

[24] Huang F, Guang P, Li F, Liu X, Zhang W, Huang W (2020). AML, ALL, and CML classification and diagnosis based on bone marrow cell morphology combined with convolutional neural network: a STARD compliant diagnosis research. Medicine (Baltimore).

[25] Joshi S, Kumar R, Dwivedi A (2021). Hybrid DSSCS and convolutional neural network for peripheral blood cell recognition system. IET Image Process.

[26] Kalaiselvi TC, Santhosh Kumar D, Subhashri KS, Siddharth S (2020). Classification of leukemia using convolution neural network. Eur J Mol Clin Med.

[27] Amin J, Sharif M, Anjum MA, Siddiqa A, Kadry S, Nam Y, Raza M (2021). 3D semantic deep learning networks for leukemia detection. Comput Mater Contin.

[28] Loddo A, Putzu L (2021). On the effectiveness of leukocytes classification methods in a real application scenario. AI.

[29] Vogado L, Veras R, Aires K, Araújo F, Silva R, Ponti M, Tavares JM (2021). Diagnosis of leukaemia in blood slides based on a fine-tuned and highly generalisable deep learning model. Sensors (Basel).

[30] Anilkumar KK, Manoj VJ, Sagi TM (2021). Automated detection of B cell and T cell acute lymphoblastic leukaemia using deep learning. IRBM.

[31] Baig R, Rehman A, Almuhaimeed A, Alzahrani A, Rauf HT (2022). Detecting malignant leukemia cells using microscopic blood smear images: a deep learning approach. Appl Sci.

[32] Claro ML, Veras RMS, Santana AM, Vogado LHS, Junior GB, de Medeiros FNS, Tavares JMR (2022). Assessing the impact of data augmentation and a combination of CNNs on leukemia classification. Inf Sci.

[33] Muhamad HA, Wahhab Kareem S, Hersh Hersh, Mohammed AS (2022). A deep learning method for detecting leukemia in real images. NeuroQuantology.

[34] Prabhakar SK, Ryu S, Jeong IC, Won DO (2022). A dual level analysis with evolutionary computing and swarm models for classification of leukemia. Biomed Res Int.

[35] Sakthiraj FSK (2022). Autonomous leukemia detection scheme based on hybrid convolutional neural network model using learning algorithm. Wirel Pers Commun.

[36] Saleem S, Amin J, Sharif M, Anjum MA, Iqbal M, Wang SH (2022). A deep network designed for segmentation and classification of leukemia using fusion of the transfer learning models. Complex Intell Syst.

[37] Houssein EH, Mohamed O, Abdel Samee N, Mahmoud NF, Talaat R, Al-Hejri AM, Al-Tam RM (2023). Using deep DenseNet with cyclical learning rate to classify leukocytes for leukemia identification. Front Oncol.

[38] Kadmin KA, Najjar HF, Waad AA, Al-Kharsan IH, Khudhair ZN, Salim AA (2023). Leukemia classification using a convolutional neural network of AML images. MJFAS.

[39] Naz I, Muhammad N, Yasmin M, Sharif M, Shah JH, Fernandes SL (2019). Robust discrimination of leukocytes protuberant types for early diagnosis of leukemia. J Mech Med Biol.

[40] Wang W, Luo M, Guo P, Wei Y, Tan Y, Shi H (2023). Artificial intelligence-assisted diagnosis of hematologic diseases based on bone marrow smears using deep neural networks. Comput Methods Programs Biomed.

